# Synthesis and Preclinical Evaluation of a Bispecific PSMA-617/RM2 Heterodimer Targeting Prostate Cancer

**DOI:** 10.1021/acsmedchemlett.4c00324

**Published:** 2024-10-18

**Authors:** Christos Liolios, Danai Bouziotis, Wiebke Sihver, Martin Schäfer, George Lambrinidis, Evangelia-Alexandra Salvanou, Ulrike Bauder-Wüst, Martina Benesova, Klaus Kopka, Antonios Kolocouris, Penelope Bouziotis

**Affiliations:** † Division of Radiopharmaceutical Chemistry, German Cancer Research Centre (DKFZ), Im Neuenheimer Feld 280, 69120 Heidelberg, Germany; ‡ Radiochemical Studies Laboratory, INRASTES, N.C.S.R. “Demokritos”, Agia Paraskevi Attikis, 15310 Athens, Greece; § Laboratory of Medicinal Chemistry, Section of Pharmaceutical Chemistry, Department of Pharmacy, National and Kapodistrian University of Athens (NKUA), Panepistimiopolis−Zografou, 15771 Athens, Greece; ∥ Institute of Radiopharmaceutical Cancer Research, Helmholtz-Zentrum Dresden-Rossendorf (HZDR), Bautzner Landstraße 400, 01328 Dresden, Germany; ⊥ Faculty of Chemistry and Food Chemistry, School of Science, Technical University Dresden, Raum 413 Bergstr. 66, 01069 Dresden, Germany; # Institute of Pharmaceutical Research & Technology (IFET), 18th km of Marathonos Avenue, 15351 Pallini, Attica, Greece; ¶ Department of Nursing & Department of Physiotherapy, School of Health and Caring Sciences, University of West Attica, Agiou Spyridonos, 12243, Egaleo, Greece

**Keywords:** prostate cancer, PSMA, GRPR, PC-3, LNCaP, ^68^Ga, ^177^Lu, cell-binding specificity, cell-internalization, radiolabeling, theranostics, molecular dynamics simulations, homology modeling, induced-fit docking

## Abstract

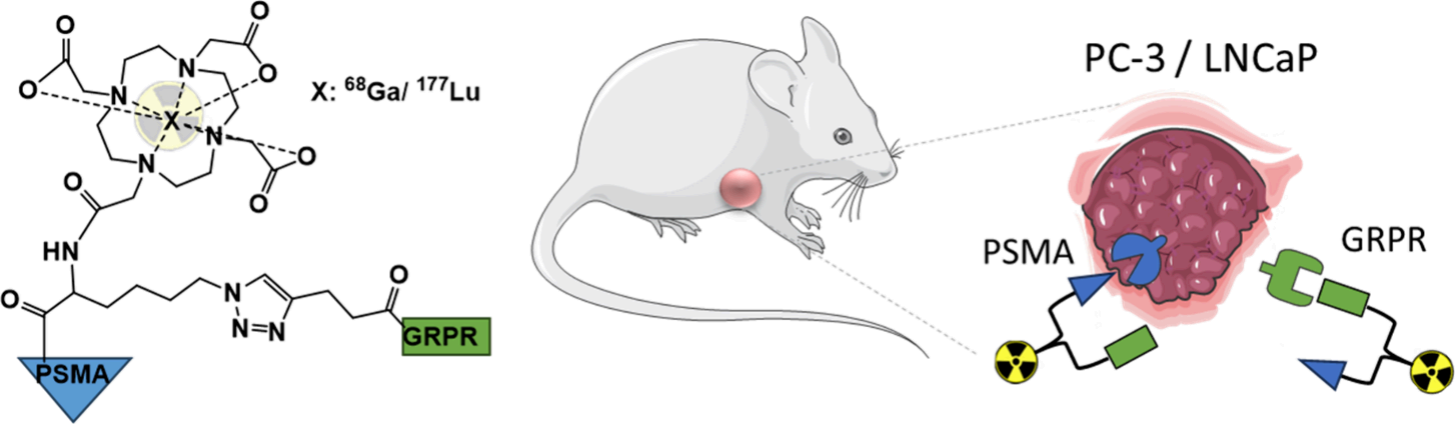

Prostate-specific membrane antigen (PSMA) and gastrin-releasing peptide receptor (GRPR) have been used for diagnostic molecular imaging/therapy of prostate cancer (PCa). To address tumor heterogeneity, we synthesized and evaluated a bispecific PSMA/GRPR ligand (**3**) combining PSMA-617 (**1**) and the GRPR antagonist RM2 (**2**) with the radiometal chelator DOTA. **3** was radiolabeled with ^68^Ga ([^68^Ga]Ga-**3**) and ^177^Lu ([^177^Lu]Lu-**3**). [^68^Ga]Ga-**3** was tested in the following PCa cell lines for receptor affinity, time kinetic cell-binding/specificity, and cell-internalization: PC-3 and LNCaP. Compared to the monomers (**1** and **2**), ligand **3** showed specific cell binding, similar receptor affinities, and higher lipophilicity, while its internalization rates and cell-binding were superior. Docking calculations showed that **3** can have binding interactions of PSMA-617 (**1**) inside the PSMA receptor funnel and RM2 (**2**) inside the GRPR. *In vivo* biodistribution studies for [^68^Ga]Ga-**3** showed dual targeting for PSMA(+) and GRPR(+) tumors and higher tumor uptake, faster pharmacokinetic, and lower kidney uptake compared to **1** and **2**

The most prevalent cancer among men globally is prostate cancer (PCa), with consistently high rates of mortality associated with the disease.^1−3^ The prostate-specific membrane antigen (PSMA)^4−7^ and gastrin-releasing peptide receptor (GRPR)^8−14^ are two targets that have been utilized for the design of numerous diagnostic and therapeutic ligands, as well as for theranostic applications in nuclear medicine (NM).^15^ PSMA or glutamate carboxypeptidase II (GCPII) is a binuclear zinc metallopeptidase protein expressed in normal prostate cells as a truncated form (PSM′) lacking the intracellular and transmembrane domains of PSMA^16^ and in PCa cells as a membrane protein, expressed on the cell surface, which eventually is overexpressed in high-grade and metastatic PCa.^6,17^ However, a decrease or loss of PSMA expression has been observed, which is often linked to the progression of PCa from an androgen-dependent to an androgen-independent stage. Additionally, there are reports of increased PSMA expression associated with androgen deprivation therapies.^18,19^ Two radiotracers, [^68^Ga]Ga-PSMA-11 and PSMA-617 (**1**, Scheme 1) (vipivotide tetraxetan), radiolabeled with ^68^Ga for imaging and with ^177^Lu (β-particle therapy) or ^225^Ac (α-particle therapy) for endoradio-therapeutic applications have tipped the scale in favor of PSMA as a target.^14,20−24^ [^68^Ga]Ga-PSMA-11 and [^177^Lu]Lu-PSMA-617 (EU approval 2022) are currently used for the diagnosis and treatment of PSMA-positive metastatic castration-resistant prostate cancer.

The membrane protein GRPR or bombesin receptor subtype 2 (BB2R), a G-coupled protein receptor (GPCR), has been also considered as a target for PCa, especially for locally recurrent PCa after brachytherapy and external beam radiotherapy.^9−11,13,14,25−27^ GRPR is overexpressed in PCa in comparison to sparse expression in normal prostate tissue, while its expression increases in well-differentiated carcinomas and is correlated with the process of prostate cells transforming into malignant neoplasms.^10,13,14,26−29^ One of the most studied GRPR ligands coupled with the DOTA chelator is the GRPR-specific antagonist DOTA-(4-amino-1-carboxymethylpiperidine)-d-Phe^6^-Gln^7^-Trp^8^-Ala^9^-Val^10^-Gly^11^-His^12^-Sta^13^-Leu^14^-NH_2_ (**2**) or RM2 (**2**) (Scheme 1). The radiolabeled [^68^Ga]Ga-**2** has entered clinical trials for the diagnosis and treatment of PCa and BCa.^10,30,31^

**Scheme 1:**
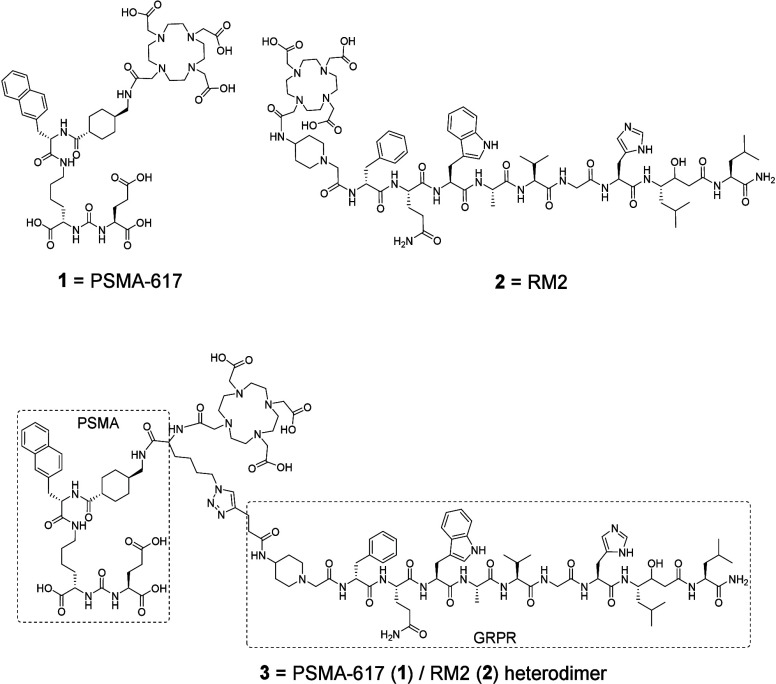
Chemical Structures of PSMA-617 (**1**), RM2 (**2**)m and Heterodimer **3**

PCa tumors show heterogeneity due to the inharmonious expression of receptors like PSMA and GRPR. Lack of detection of either receptor’s expression may significantly reduce the image quality and detection ability of the PCa-associated lesion.^32−34^ A novel strategy that could improve the sensitivity of PET imaging for biochemically recurrent PCa and enhance the clinical relevance of the diagnostic assessment is the development of heterodimeric molecules combining two specificities: PSMA and GRPR.^20,35−38^ A low molecular weight heterodimer consists of two covalently linked peptides or peptidomimetics combining specificities for two different antigens/epitopes, while a chelator group (*i.e.*, the open chelator HBED-CC,^39,40^ NOTA,^41,42^ DOTA,^43^ or DO3A^44^) for radiometal complexation is usually included in the structure.^35,42,45^

Previous studies from our group with heterodimers utilized the Lys-CO-Glu-OH pharmacophore (PSMA) and the GRPR agonist H_2_N-PEG_2_-[d-Tyr^6^,β-Ala^11^,Thi^13^,Nle^14^)BN(6–14) covalently connected with the HBED-CC chelator linked via its two carboxylic groups with either one of the pharmacophores.^39,40^ The BN analogue used in those cases was structurally relevant to the peptidase-resistant BN analogue that was used in the clinically tested [^68^Ga]GaBZH_3_ (BZH_3_ = DOTA-PEG_2_-[d-Tyr^6^,β-Ala^11^,Thi^13^,Nle^14^]BN(6–14) amide).^46,47^ These heterodimers showed high affinity values for both PSMA and GRPR targets, (*i.e.*, PC-3, 4.40–9.00 nM; LNCaP, 17.4–42.4 nM) with high uptake and specific tumor uptake in LNCaP and AR42J xenographs.^39,40^ The addition of the (HE)_*n*_ (*n* = 1–3) amino acid spacer between the HBED-CC chelator and the PSMA pharmacophore (HE_0–3_) reduced the kidney (73.25–87.57% IA/g, 1 h pi) uptake and in some cases improved the tumor uptake, resulting in higher values than the respective monomers.^40^

Another heterodimeric radiotracer, [^68^Ga]Ga-iPSMA-BN, consisted of the iPSMA (Lys(NaI)-urea-Glu-OH; Nal = 3-(1-naphthyl)-l-alanine) oligopeptide and the Lys^3^-BN(1–14) peptide agonist, both linked to a DOTA chelator.^43^ Heterodimer [^68^Ga]Ga-iPSMA-BN showed superiority against each monomer, [^68^Ga]Ga-iPSMA and [^68^Ga]Ga-BN, in both cell lines (LNCaP and PC-3 cells) and animal models.^43^ The ligand was also labeled with ^177^Lu and evaluated with *ex vivo* biodistributions and Micro-SPECT/CT imaging studies in xenographed mice, which proved the positive influence of the heterobivalent effect.^48^ Furthermore, pharmacokinetics and dosimetry data for [^68^Ga]Ga-iPSMA-BN were collected in a study involving four healthy volunteers, revealing specific uptake in the pancreas, which expresses GRPR, and the salivary glands, which express PSMA.^49^

Three additional bispecific heterodimers based on the antagonistic peptide RM26 (d-Phe^6^-Gln^7^-Trp^8^-Ala^9^-Val^10^-Gly^11^-His^12^-Sta^13^-Leu^14^-NH_2_) for GRPR and PSMA-617 (**1**) were developed^50^ using the general structure PSMA-617-X-triazolyl-Tyr-PEG_2_-RM26 (X = 0, PEG_2_, (CH_2_)_8_), where the two pharmacophores were linked via the spacer X-triazolyl-Tyr-PEG_2_. The resulting heterodimers were radio-iodinated (^125^I) and evaluated *in vitro* and *in vivo* in PC-3 (IC_50_ = 6.0–20.0 nM) and LNCaP and PC-3 PIP (IC_50_ = 80.0–100.0 nM) (PSMA/GRPR positive) xenographs. All resulting heterodimers showed binding specificity, cellular retention, and affinity, while among them [^125^I]I-PSMA-617-PEG_2_-triazolyl-Tyr-PEG_2_-RM26 presented the highest values regarding cancer cell uptake and higher tumor accumulation (PC-3, 4.3% ID/g, PC-3 PiP 10% ID/g at 1 h pi). However, it also showed high kidney radioactivity values (66% ID/g and 56% ID/g, respectively).^50^

A heterodimer based on the pharmacophores RM26 (GRPR) and DUPA (PSMA) and the NOTA chelator was labeled with ^111^In and ^68^Ga.^41^ The ligand showed affinity toward GRPR (IC_50_ = 4 ± 1 nM) and PSMA (IC_50_ = 824 ± 230 nM), which was less than the monomers (10-fold GRP, 5-fold PSMA). Despite its low PSMA affinity, it exhibited tumor uptake in the PC-3-PIP-xenografted mice at 1 h pi (^68^Ga labeling, 8 ± 2% ID/g; ^111^In labeling, 12 ± 2% ID/g), along with less kidney uptake (^68^Ga labeling, 6.6 ± 0.8% ID/g; ^111^In labeling, 10 ± 2% ID/g).^41^ The lower kidney uptake was possibly due to its low PSMA affinity, considering the fact the kidneys naturally express PSMA receptors.^51^

Three additional heterodimers, based on the pharmacophore RM26 (GRPR)/DUPA (PSMA) connected to the NOTA chelator via a PEG and an Aoc-Phe linker, were investigated after labeling with ^111^In. Among the three heterodimers, [^111^In]In-BQ7812, with Phe and the short PEG linker, demonstrated the best affinity toward PSMA (IC_50_ = 102 ± 80 nM) and thus was selected for biodistribution studies,^42^ where it showed specific uptake (1 h pi, 16.10 ± 2.96% ID/g) for the PC-3-pip tumor (PSMA+/ GRPR+) and high kidney uptake (64.87 ± 27.26% ID/g).^42^ The same ligand was labeled with ^68^Ga [^68^Ga]Ga-BQ7812 in a later study,^52^ where it showed a similar pharmacokinetic profile (tumor, 10.4 ± 1.0% ID/g; kidneys, 45 ± 16% IA/g).

In the present study, we synthesized a heterodimer (**3**) using the core structures of PSMA-617 (**1**) and RM2 (**2**) ligands and the DOTA chelator (Scheme 1). Synthesis of pharmacophores **1′** and **2′** was accomplished using SPPS on 2-chloro-trytyl resin and rink amide resin, respectively, as outlined in Scheme 2 according to standard Fmoc peptide synthesis protocols.^40,53^ The PSMA-specific azido-PSMA-617 analogue (**1′**) and GRPR-specific alkyno-RM2 analogue (**2′**) were each cleaved from the resin with TFA/TIPS/H_2_O, purified by RP-HPLC, and analyzed with MALDI-MS (see SI, Figure S1, Table S1). In the next step, they reacted with CuAAC, and the intermediate conjugate was coupled with DOTA-mono-*N*-hydroxysuccinimide ester (DOTA-NHS-ester) to form heterodimer **3**, which was purified with RP-HPLC and analyzed with MALDI-MS (see SI, Figure S1, Table S1). The universal DOTA chelator that was utilized replaced the HBED-CC chelator of previously developed heterodimers^39,40^ in order to provide theranostic potential, since [^68^Ga[Ga]-HBED-CC] can only be utilized for diagnosis.

**Scheme 2:**
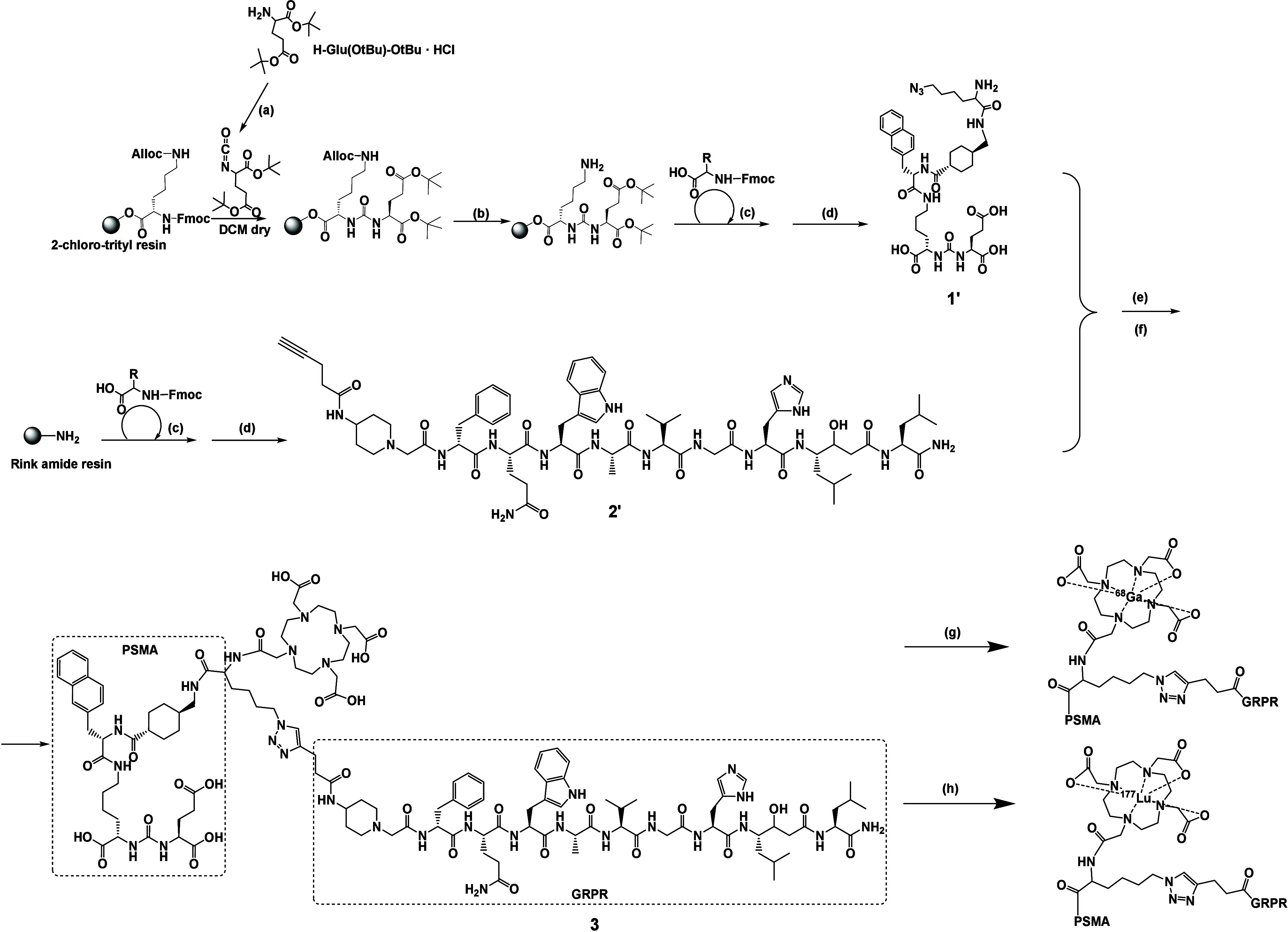
Chemical Synthesis of PSMA-Specific **1′**, GRPR-Specific **2′** , and Heterodimeric Conjugate **3** (a) Triphosgene, DIPEA, DCM (°C). (b) Pd(PPh_3_)_4_, morpholine, DCM (dry). (c) Amino acid (a.a.) and amino acid derivatives (6-azido-l-lysine, 4-pentynoic acid) coupling: a.a./DIPEA/HBTU (4.0:4.0:3.9 equiv). Fmoc deprotection: 40% piperidine in DMF. (d) Cleavage mixture: TFA/TIPS/H_2_O 95:2.5:2.5 (v/v/v). (e) CuAAC reaction (4 equiv of CuSO_4_, 4 equiv of Na-ascorbate). (f) DOTA-NHS, EDC, PBS (pH = 8.5). (g) [^68^Ga]Ga, Hepes buffer (0.25 M), pH = 4.0, 95 °C, 30 min. (h) [^177^Lu]LuCl_3_, Na–Ac buffer (400 nM), pH = 5.0, 98 °C, 25 min.

Heterodimer **3** and the two monomers, PSMA-617 (**1**) and RM2 (**2**) (controls), were radiolabeled with (i) the PET diagnostic ^68^Ga [half-life (*T*_1/2_) = 68 min; maximum energy of positrons (b1) = 1.9 MeV [88%]) in HEPES (*N*-2-hydroxyethylpiperazine-*N*-2-ethane sulfonic acid) buffer, resulting in [^68^Ga]Ga-PSMA-617, [^68^Ga]Ga-RM2, and [^68^Ga]Ga-**3**, and (ii) with the therapeutic ^177^Lu (*T*_1/2_ = 6.71 d; maximum energy of electrons [β^–^] = 497 keV [79%]; energy of photons (γ) = 113 keV [6%]; roentgen radiation (x) = 208 keV [11%]) in sodium acetate (Na–Ac) buffer, resulting in [^177^Lu]Lu-PSMA-617, [^177^Lu]Lu-RM2, and [^177^Lu]Lu-**3**. During the radio RP-HPLC analysis, PSMA-617 (**1**) was eluted first and the heterodimer **3** was eluted last, which was in accordance with the size of each ligand (see SI, Figure S1). Radiochemical purity in all cases was above 95%, while radiochemical yield was over 90%, a crucial requirement for possible future application as theranostic agents.

The lipophilicities of all compounds, *i.e.*, [^68^Ga]Ga-PSMA-617, [^68^Ga]Ga-RM2, [^68^Ga]Ga-**3**, [^177^Lu]Lu-PSMA-617, [^177^Lu]Lu-RM2, and [^177^Lu]Lu-**3**, were determined by measuring their equilibrium distributions in a two-phase system consisting of *n*-octanol and phosphate buffer solution (PBS) with pH 7.4. In all cases, negative logD values were observed, showing the preference of all compounds for the water phase; however, in both cases the monomers were more hydrophilic than the heterodimer **3**, which also showed logD values that were negative but closer to zero. This agreed with previously mentioned results from the radio RP-HPLC analysis (see SI, Table S2).

Docking calculations were performed for the PSMA-617 part and the RM2 part of the heterodimer (**3**) in the binding area of PSMA^54^ or GRPR, respectively. The active site of PSMA is bordered by two binding regions,^5^ with Arg210 or K699 in one region interacting through their side chain guanidinium or ammonium groups, respectively, with the side chain carboxyl of glutamate in the Glu-urea-Lys pharmacophore of PSMA-617 and the arginine patch, which contains Arg463, Arg534, and Arg536, in the other pocket interacting with Lys’s carboxylate. The urea group carbonyls bind the two zinc ions.^55,56^ The chelator can be stabilized with ionic hydrogen bonding interactions with cationic amino acids in the entrance of the funnel, which has ∼20 Å length, *e.g.*, with R610 or K514 (Figure 1A and B), and the RM2 lies outside the PSMA channel (Figure 1A). The structure of the GRPR in complex with Gαq and the peptide-agonist [d-Phe^6^,β-Ala^11^,Phe^13^,Nle^14^)Bn(6–14)] with PDB ID 7W40(57) was recently solved with electron cryo-microscopy (cryo-EM). This structure was used as a template for the docking calculations. As a BN antagonist, the GPCR-bound part of heterodimer **3**, *i.e.*, the RM2 part, binds tightly toGRPR. Thus, its C-amidated end forms hydrogen bonding interactions with critical amino acids^57^ at the bottom of the binding area, *e.g.*, Q120^3.32^ and R308^7.39^ (Figure 1C), the main chain is stabilized inside the GPCR bundle through numerous hydrophobic interactions, and the peptide antagonist interacts with ECL2 at the extracellular regions. The chelator and PSMA-617 parts of **3** lie in the extracellular part of the GPCR (see Figure 1D and SI).

**Figure 1:**
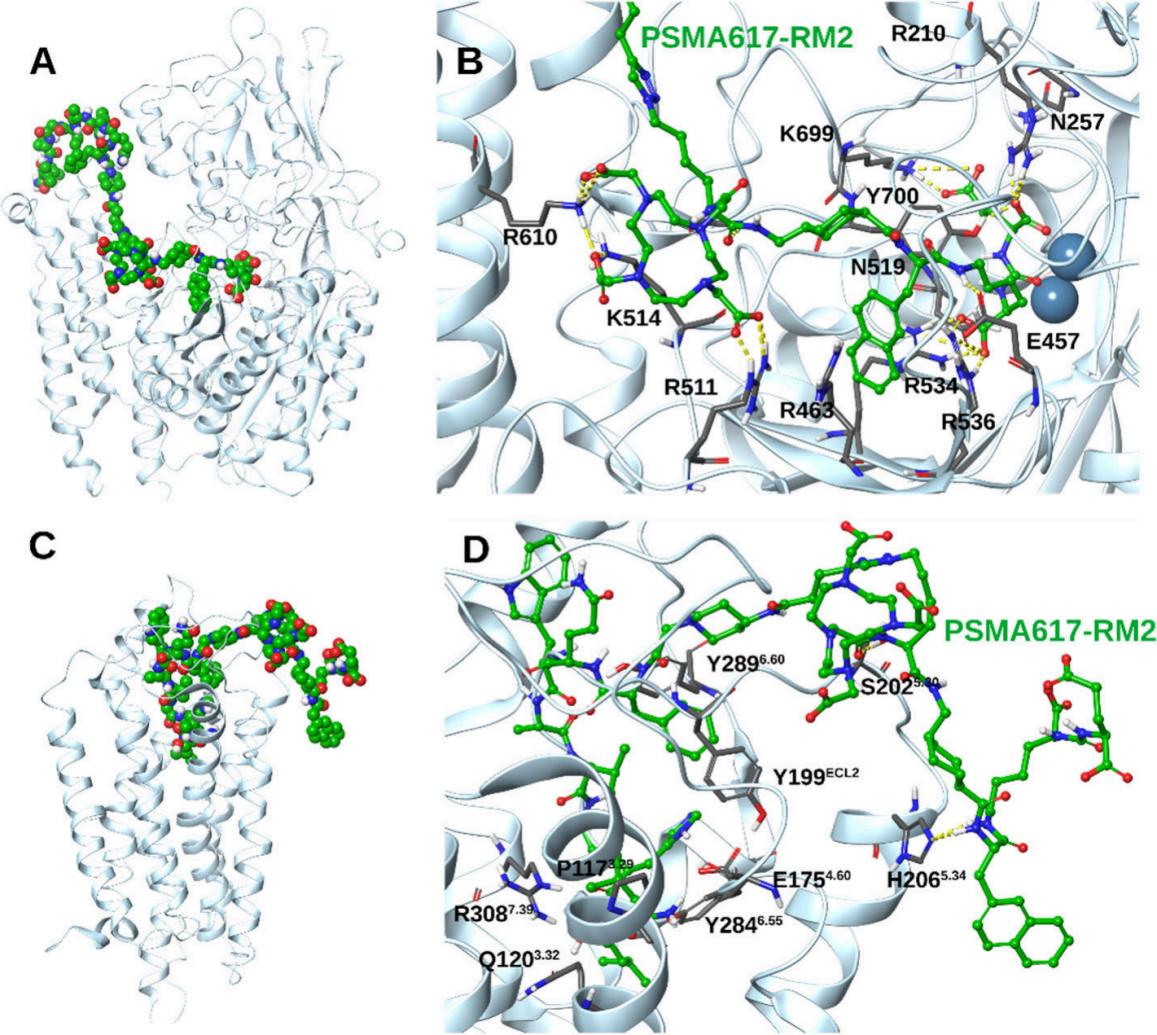
Results from docking calculations of heterodimer **3** (A, B) inside the PSMA receptor and (C, D) inside the BB2R. (A) Docked PSMA-617 part of **3** inside the PSMA funnel; the zoomed-in view shows the Glu-urea-Lys-linker-chelator binding. (C, D) Binding of RM2 peptide part of heterodimer **3** inside the GPCR BB2R (ligand carbons, green; oxygen, red; nitrogen, blue; polar hydrogen: white, the receptor is shown with a light blue cartoon representation).

The inhibition potency (IC_50_) of heterodimer **3** was determined by a cell-based competitive assay (*C* = 0–5000 nM) with LNCaP (PSMA+, GRPR−) and PC-3 cells (PSMA–, GRPR+) (Figure 2). The affinity (IC_50_) of PSMA-617 (**1**) for PSMA was 6.41 nM, and that of **3** was 21.41 nM, while for GRPR the affinity of RM2 (**2**) (IC_50_ = 45.59 nM) and that of **3** (IC_50_ = 43.93 nM) were almost equal (See S.I. Table S3). In summary, after *in vitro* testing in LNCaP and PC-3 cells, heterodimer **3** showed similar affinities for the PSMA receptor and GRPR compared to both control monomers PSMA-617 (**1**) and RM2 (**2**), respectively.

**Figure 2:**
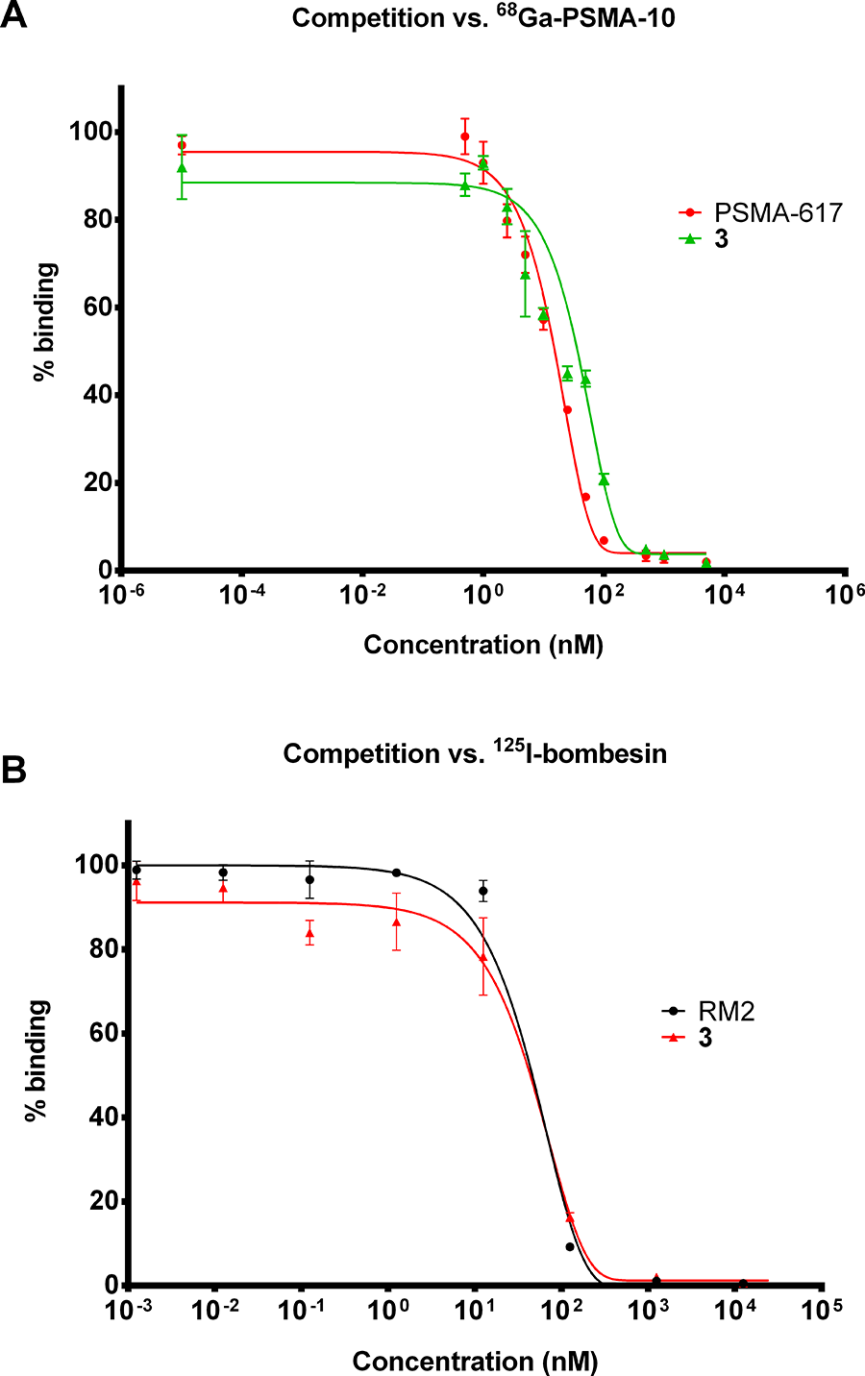
Competitive binding curves plotted using various concentrations (*C* = 0–5000 nM) of **3** and controls (A) PSMA-617 (**1**) against [^68^Ga]Ga-PSMA-10 (standard, IC_50_ = 3.8 ± 1.8 nM, *C* = 0.75 nM) and (b) RM2 (**2**) against ^125^I-bombesin (standard, IC_50_ = 0.4 nM, C = 50 pM). Each value was measured in quadruplicate.

Time kinetic data for heterodimer [^68^Ga]Ga-**3** and the controls, [^68^Ga]Ga-PSMA-617 (LNCaP) and [^68^Ga]Ga-RM2, (PC-3), were investigated in the time range of 0–120 min, while blocking studies were also conducted (see S.I., Figure S3). Heterodimer [^68^Ga]Ga-**3** presented specific cell binding in both cell lines, *i.e.*, LNCaP and PC-3, while specificity was proved after minimization of cell-bound radioactivity during the blocking experiments (Figure S3). Heterodimer [^68^Ga]Ga-**3** in all cases presented higher cell uptake in comparison to that of the monomers (controls) (Figure S3).

The above ^68^Ga-labeled tracers were also tested in LNCaP and PC-3 cells at 37 and 4 °C (45 min incubation time) to determine the fractions of surface-bound and internalized radio-ligand (Figure 3).

**Figure 3:**
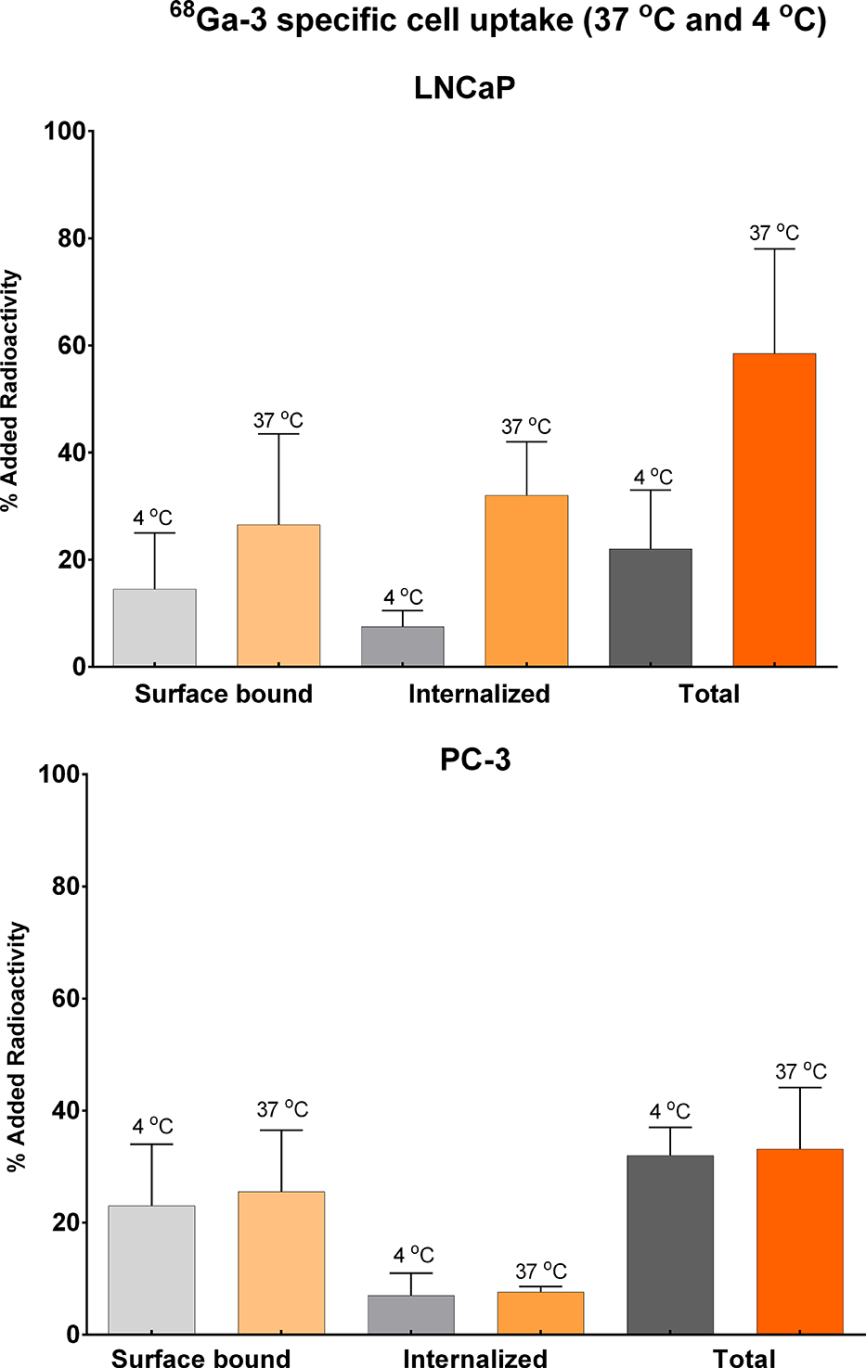
Specific cell-bound radioactivity (surface, internalized, and total) for [^68^Ga]Ga-**3** at 37 and 4 °C in (A) LNCaP and (B) PC-3 cells. Results are expressed as the percentage of the added radioactivity for 10^6^ cells (mean values % ID/g ± SD, *N* = 3–4).

The majority of [^68^Ga]Ga-**3** was surface-bound. The amount of total cell-bound [^68^Ga]Ga-**3**, summing up the internalized and surface-bound fractions for LNCaP and PC-3 cells, was in both cases higher than or comparable to that the corresponding monomers [^68^Ga]Ga-PSMA-617 (**1**) and [^68^Ga]Ga-RM2 (**2**) (Figure 4). As expected, at 4 °C energy-dependent internalization was minimized, while the surface-bound fraction remained practically the same (Figure 3). In addition, the percentage of bound ligand **3** for the LNCaP cells was much higher than that for the PC-3 cells. All the above *in vitro* assays further established the specificity of ligand [^68^Ga]Ga-**3** for both PSMA and GRPR, while its total internalization rates and cell binding showed superiority over both monomers.

**Figure 4:**
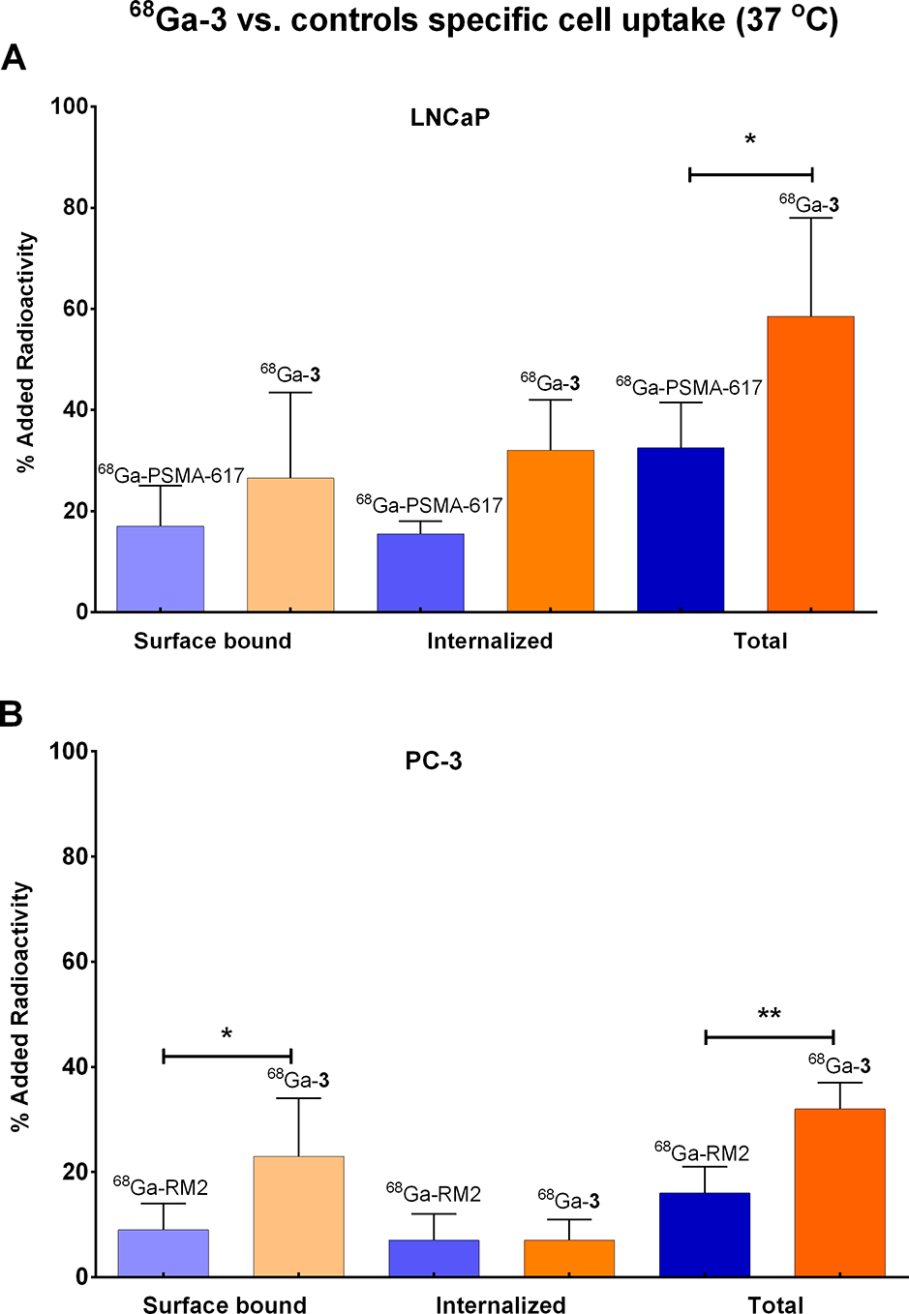
Comparison of [^68^Ga]Ga-**3** with the controls (A) [^68^Ga]Ga-PSMA-617 and (B) [^68^Ga]Ga-RM2. Results are expressed as the percentage of the added radioactivity for 10^6^ cells (mean values % ID/g ± SD, *N* = 3–4). Statistical differences are noted with * above the bars (one-way Anova, α = 0.1, *p < 0.05, **p < 0.01).

The pharmacokinetic profile and tumor targeting ability of the radiolabeled heterodimer [^68^Ga]Ga-**3** were examined with organ distribution experiments (30, 60, and 120 min pi) in Swiss albino mice bearing PC-3 (Figure 5A) and LNCaP (Figure 5B) tumors. The heterodimer [^68^Ga]Ga-**3** showed fast blood clearance, while it was mainly excreted via the kidneys into the urinary bladder. Tumor uptake was higher for the LNCaP tumors than for PC-3 tumors, which can be attributed to the higher expression of PSMA in LNCaP cells in comparison to that of GRPR in PC-3 cells (levels of expression: PSMA, 1.26–1.8 × 10^5^ per LNCaP; GRPR, 9.7 × 10^4^ per PC-3 cell)^58−60^ In addition, the amount of [^68^Ga]Ga-**3** inside the LNCaP tumors did not degrade as fast as the one in the PC-3 tumors, possibly because of the higher rates of internalization for the PSMA ligand–receptor complexes compared to the GRPR antagonist complexes.

**Figure 5:**
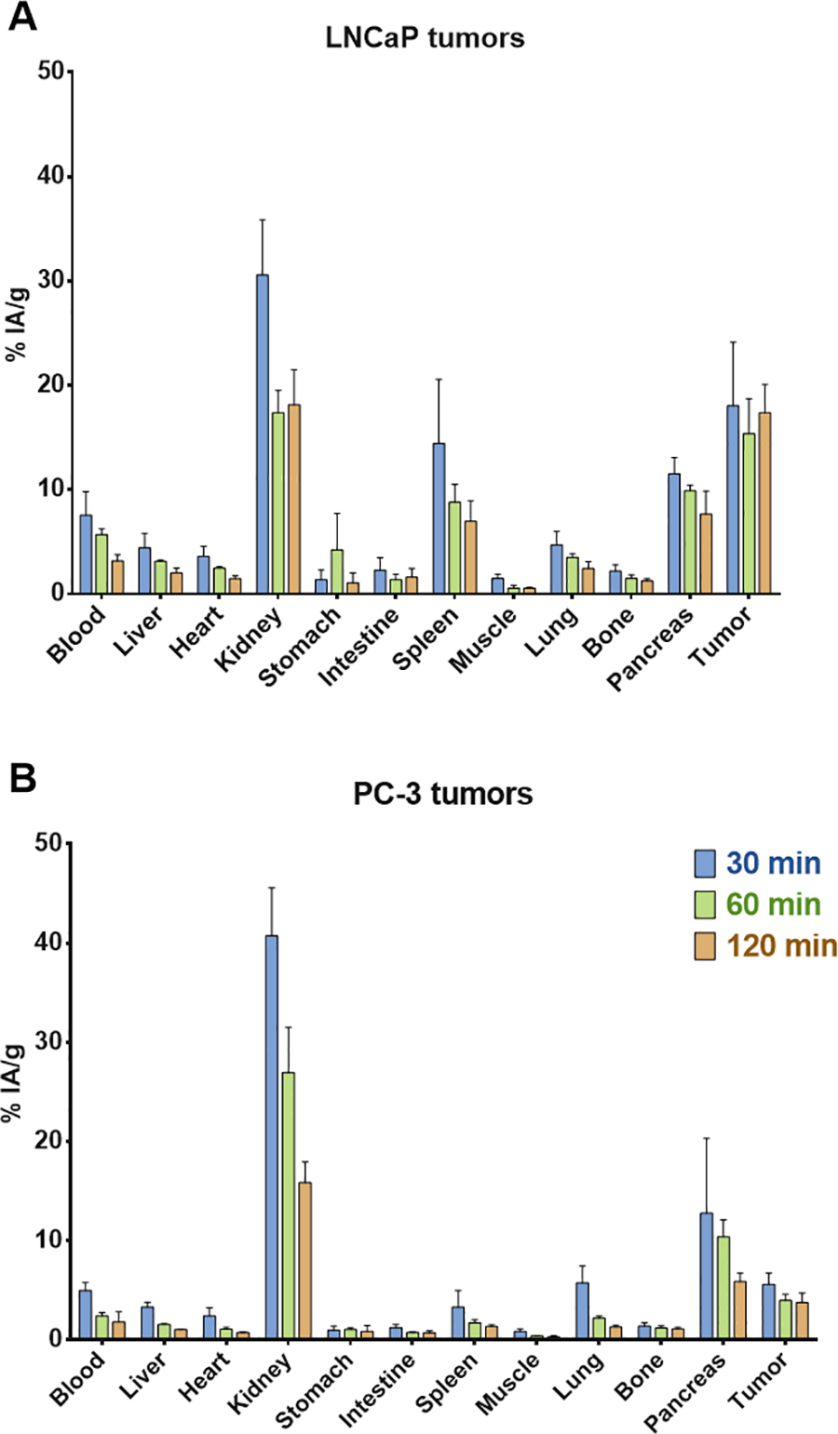
Biodistribution results expressed as % IA/g for [^68^Ga]Ga-**3** in nude mice bearing (A) LNCaP and (B) PC-3 tumors at three different time points 30, 60, and 120 min pi.

Considering the increased expression of PSMA in the kidneys, the kidney uptake was much lower than that in other heterodimers we previously developed, which ranged between 66% and 122% IA/g.^39,40^ The observed off-target uptake in the pancreas was due to the normal expression of GRPRs in this tissue; however, pancratic uptake degraded with time at a faster rate than the radio-activity located in the tumors. Such off-target accumulation has also been described for previously studied heterodimers, *e.g.*, [^68^Ga]Ga-iPSMA-BN.^43^ In general, [^68^Ga]Ga-**3** showed dual targeting of PSMA-positive and GRPR-positive tumors and mainly renal clearance from the body.

In comparison to literature data regarding the biodistribution of ^68^Ga-**3** and the monomers (1 h pi), a higher LNCaP tumor uptake was observed (^68^Ga-**3**, 15.36 ± 3.34% ID/g; ^68^Ga-**1**, 8.47 ± 4.09% ID/g^21^) in combination with lower kidneys uptake (^68^Ga-**3**, 17.38 ± 2.14; ^68^Ga-**1**, 113.3 ± 24.4% ID/g^21^), while the opposite results were observed for the PC-3 tumors (^68^Ga-**3**, 3.96 ± 0.63% ID/g; ^68^Ga-**2**, 14.11 ± 1.88% ID/g^30^) and for the kidneys (^68^Ga-**3**, 26.94 ± 4.55% ID/g; ^68^Ga-**2**, 3.34 ± 0.54% ID/g^30^). ^68^Ga-**3** showed higher tumor uptake than previously published ^68^Ga-labeled heterodimers using RM26 and DUPA pharmacophores (1 h pi, PC-3-PIP tumor, 8 ± 2% ID/g^41^ and 10.4 ± 1.0% ID/g^52^). However, such a direct comparison is not accurate due to the different tumor model used.

The differences in the wash out profile of ^68^Ga-**3** in the two different animal models could be attributed to its accumulation in the different kinds of tumors (LNCaP and PC-3) in combination with its accumulation in normal organs expressing PSMA or GRPR receptors. For example, in the mice bearing LNCaP tumors (PSMA+, GRPR−), a lower % ID/g was observed for the kidneys (30 and 60 min) than in the kidneys of the mice bearing PC-3 tumors. The reason for this is that the kidneys express PSMA receptors and, in the first case, less ^68^Ga-**3** was available for the kidneys due to ^68^Ga-**3** accumulation in the LNCaP tumor. Considering also that kidney–urinary bladder–urine was the major route of clearance for ^68^Ga-**3**, kidney PSMA receptors affected the washout profile, resulting in slightly higher values for blood % ID/g.

Tumor/tissue ratios for the two tumor animal models (PC-3 and LNCaP) showed increasing contrast for most of the tissues, *i.e.*, blood/heart, muscle, spleen, intestines, and lungs, over time, which was much more intense for the LNCaP tumors due to the higher uptake of ^68^Ga-**3** (Figure 6). Tumor/kidneys ratios were very low due to the reasons mentioned previously. However, the tumor/pancreas ratio also showed a trend of increasing over time despite the fact that pancreas expresses GRPRs.

**Figure 6:**
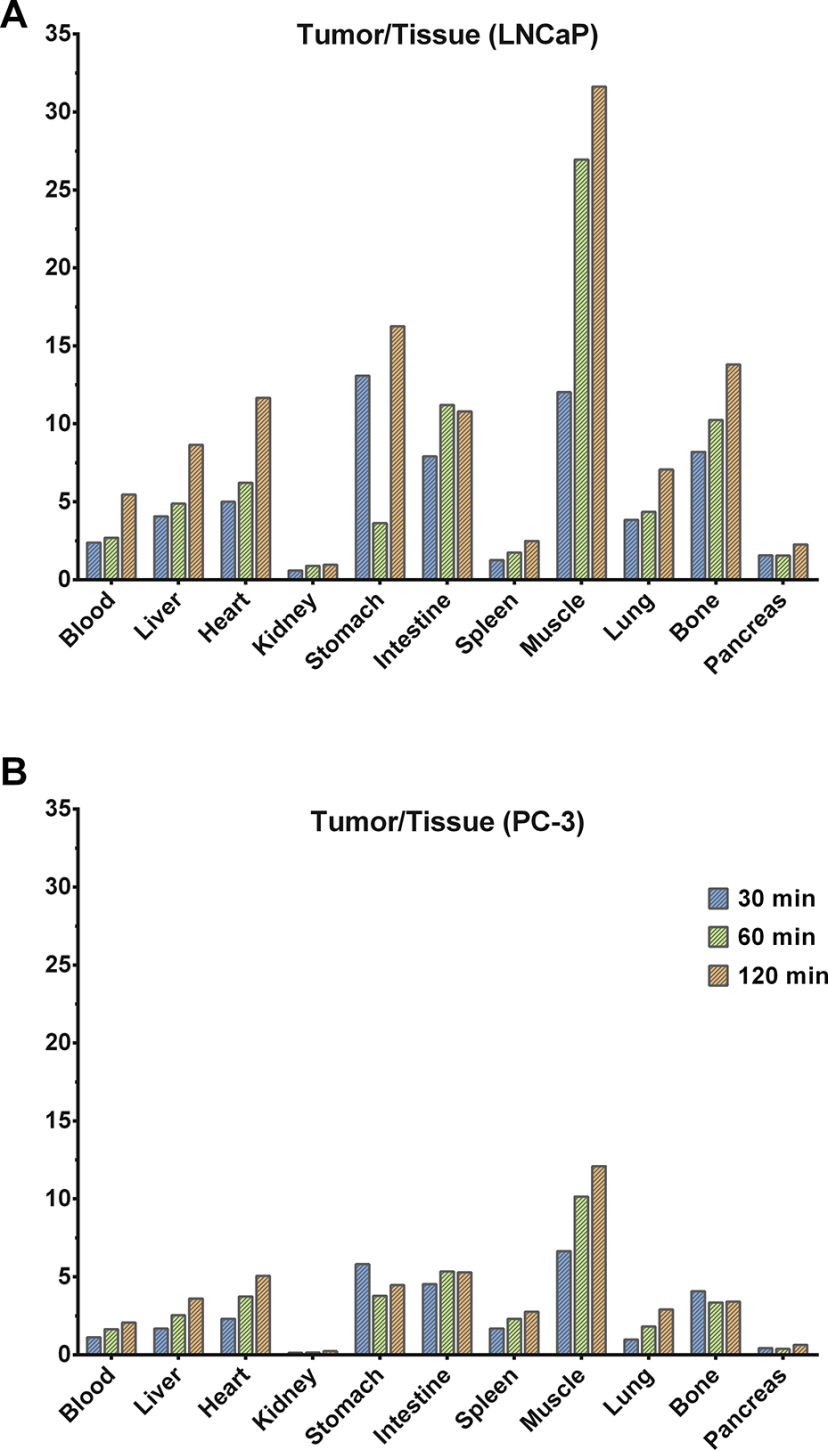
Tumor/tissue ratios for [^68^Ga]Ga-**3** in nude mice bearing (A) LNCaP and (B) PC-3 tumors at three different time points 30, 60, and 120 min pi.

In conclusion, heterodimer **3** combining the PSMA-specific scaffold of PSMA-617 and the GRPR scaffold of RM26 with the DOTA chelator demonstrated simple and high yielding radiolabeling for both ^68^Ga/^177^Lu. Ligand **3** showed similar affinities for PSMA and GRPR receptors in comparison to control monomers (PSMA-617 and RM2, respectively), which were further supported by the results of the docking calculations. *In vitro* assays established the specificity of [^68^Ga]Ga-**3** for PSMA/GRPR, while total internalization rates and cell binding showed its superiority over both monomers. *In vivo*, the high tumor accumulation of [^68^Ga]Ga-**3** in combination with its low off-target organ radio-activity make it suitable for further studies as a PET-imaging agent, while its combination with [^177^Lu]Lu-**3** could result in a theranostic pair. These results were a proof-of-concept justifying the future investigation of **3** with *in vivo* imaging experiments in order to investigate and possibly improve its pharmacokinetics, *i.e.*, with the inclusion of various linkers.

## Experimental Procedures

See also the SI for a detailed analysis of the material and methods.

### Safety

Caution: Due to radiation emission during the handling of radionuclides, *e.g.*, ^68^Ga ([^68^Ga]Ga-**3**) and ^177^Lu ([^177^Lu]Lu-**3**), all studies were conducted in a radiation laboratory equipped with HEPA filtered hoods, appropriate radiation shielding, and dosimetry safety measurements for working personnel. Caution: Triphosgene, also named BTC, used in the synthesis of **1′** is toxic and fatal if inhaled.

## Supporting Information Available

General materials and methods, compound preparation, radiolabeling, determination of lipophilicity, general cell culture and cell assays, determination of binding affinity in PC-3 and LNCaP cells, time kinetic cell binding, internalization in PC-3 and LNCAP cells, biodistribution, and docking calculations (PDF)

## Abbreviations

Aoc: 8-aminooctanoic acid
CuAAC: copper-catalyzed click chemistry
DUPA: 2-[3-(1,3-dicarboxypropyl)ureido]pentanedioic acid
GCPII: glutamate carboxypeptidase II
GPCR: G protein-coupled receptors
GRPR: gastrin releasing peptide receptor
GUI: graphical user interface
MD: molecular dynamics
NM: nuclear medicine
Nle: norleucine
PET: positron electron tomography
PCa: prostate cancer
pi.: post-injection
PSMA: prostate-specific membrane antigen
Phe: phenylalanine
POPC: 1-palmitoyl-2-oleoyl-*sn*-glycero-3-phosphatidylcholine
SPECT: single-photon emission computerized tomography
SPPS: solid-phase peptide synthesis
Thi: 3-thienylalanine,
