# Supplementary material for: Synthesis and Preclinical Evaluation of a Bispecific PSMA-617/RM2 Heterodimer Targeting Prostate Cancer
Source: ACS Med Chem Lett. 2024 Oct 18;15(11):1970–8. doi: 10.1021/acsmedchemlett.4c00324 (PMC11571012; doi:10.1021/acsmedchemlett.4c00324)
Supplement: Supplementary file 1 — ml4c00324_si_001.pdf [file ml4c00324_si_001.pdf]

SUPPORTING INFORMATION

Synthesis and Preclinical Evaluation of a Bispecific PSMA-617 / RM2 Heterodimer Targeting Prostate Cancer

Christos Liolios,*[1,2,6,7] Danai Bouziotis,[2] Wiebke Sihver,[4] Martin Schäfer,[1] George Lambrinidis,[3] Evangelia-Alexandra Salvanou,[2] Ulrike Bauder-Wüst,[1] Martina Benesova,[1] Klaus Kopka,[4,5] Antonios Kolocouris,[3] Penelope Bouziotis[2]

[1]Division of Radiopharmaceutical Chemistry, German Cancer Research Centre (DKFZ), Im Neuenheimer Feld 280, 69120 Heidelberg, Germany
[2]Radiochemical studies Laboratory, INRASTES, N.C.S.R. "Demokritos", Agia Paraskevi Attikis, 15310 Athens, Greece
[3]Laboratory of Medicinal Chemistry, Section of Pharmaceutical Chemistry, Department of Pharmacy, National and Kapodistrian University of Athens (NKUA), Panepistimiopolis−Zografou, 15771 Athens, Greece
[4]Institute of Radiopharmaceutical Cancer Research, Helmholtz-Zentrum Dresden-Rossendorf (HZDR), Bautzner Landstraße 400, 01328 Dresden, Germany
[5]Faculty of Chemistry and Food Chemistry, School of Science, Technical University Dresden, Raum 413 Bergstr. 66, 01069, Dresden, Germany
[6]Institute of Pharmaceutical Research & Technology, 18th km of Marathonos Avenue, 153 51, Pallini, Attica, Greece
[7]Department of Nursing & Department of Physiotherapy, School of Health and Caring Sciences, University of West Attica, Agiou Spyridonos, 12243, Egaleo, Greece.

Corresponding author:
Christos Liolios,
e-mail: c.liolios@ifet.gr,
T: +30 2132002539,
current address: Institute of Pharmaceutical Research & Technology, 18th km of Marathonos Avenue, 153 51, Pallini, Attica, Greece

Table of Contents

Chemistry S2
General S2
Compound preparation S2
Radiolabeling S4
Determination of lipophilicity S4
Biological testing S5
Cell Culture & cell assays S5
Determination of Binding Affinity in LNCaP and PC-3 cells S5
Time Kinetic Cell Binding S6
Internalization Experiments in PC-3 and LNCaP Cells S8
Biodistribution S8
Docking calculations S8

References S9

Chemistry

General

All commercially available chemicals were of analytical grade (over 95% pure by HPLC) and were used without further purification. The chemical suppliers were: Sigma-Aldrich (Taufkirchen, Germany) and Merck (Darmstadt, Germany), unless otherwise indicated. Protected amino acids (a.a.) and resins were supplied from Novabiochem (Merck, Darmstadt, Germany) and IRIS Biotech. (Marktredwitz, Germany). For all reaction products the chemical purity was greater than 95% as determined by RP-HPLC.

The following RP-HPLC systems were used: (a) for purifications, VWR International, La Prep UV/vis detector P314, pumps P110, column: Nucleodur Sphinx RP, 5 µm VP 250/21 (MACHEREY-NAGEL GmbH & Co. KG, Düren, Germany), gradient used (A-B): 10−90% B in 20 min, flow: 20 mL/min, (A) 0.1% TFA in H_2O and (B) 0.1% TFA in AcCN. (b) for analysis: Agilent 1100 series, multiwavelength detector (MWD), *γ*-detector (Bioscan; Washington, USA), analytical column: Chromolith RP-18e (4.6 mm × 100 mm; Merck, Darmstadt, Germany), gradient used (A−B) 0−100% B in 6 min, flow: 4 mL/min. Mass spectrometry was performed with a MALDI-MS Daltonics Microflex system (Bruker Daltonics, Bremen, Germany). Full-scan single mass spectra were obtained by scanning m/z = 200−4000 (2,5- dihydroxybenzoic acid in H_2O/AcCN 1:1 was used as matrix). For all *in vitro* and *in vivo* experiments, a NaI (TI) γ counter (Packard Cobra II, GMI, Minnesota, USA) was used for the measurement of radioactive probes.

Compound preparation

Molecules **1´** and **2´** were synthesized on a 2-chloro-trityl resin (C = 1.22 mmol/g, 100-200 mesh), Rink amide resin (200−400 mesh) (Merck, Darmstadt). Amino acid coupling was according to standard Fmoc peptide synthesis protocols (amino acid/HBTU/DIPEA, 4.0/3.9/4.0 equiv, 30 min, rt), while Fmoc was removed by washing resin with dimethylformamide (DMF)/piperidine (1:1, v/v, 3x 1.0 mL/ 5 min). At the final step the peptides were cleaved from the resin with the following mixture TFA/TIPS/H_2O (95/2.5/2.5, v/v/v), precipitated in ice-cold (0 °C) diethyl ether, and purified with semipreparative HPLC (**Figure S1**, RP-HPLC analysis of **1´** and **2´**). The coupling of the chelator DOTA-NHS (1.5 equiv.) (2,2′,2”-(10-(2-((2,5-dioxopyrrolidin-1-yl)oxy)-2-oxoethyl)-1,4,7,10-tetraazacyclododecane-1,4,7-triyl)triacetic acid, CheMatech, Dijon, France) was accomplished with EDC (1.5 equiv) (1-ethyl-3-(3-dimethylaminopropyl)carbodiimide hydrochloride) in PBS (pH= 8.5) (**Scheme 1**). Compounds **1´**, **2´** and **3** were purified by semipreparative RP-HPLC and analyzed RP-HPC (**Figure S1**) and with MALDI-MS (**Table S1**) which showed over 95% purity.

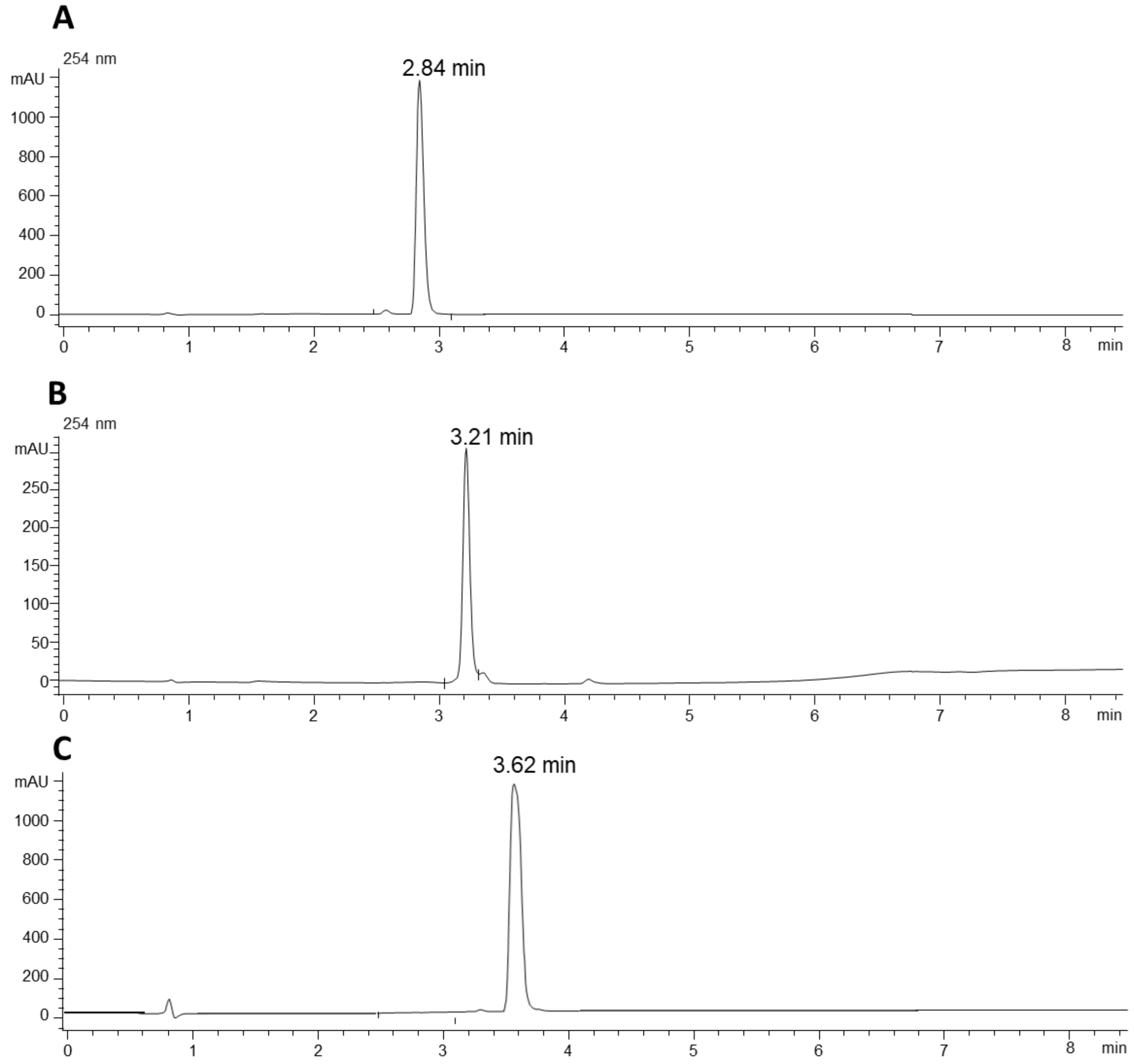

Figure S1
Analytical RP-HPLC chromatographs (n =254 nm) for pure PSMA and GRPR intermediate compounds (A) **1´** and (B) **2´**, respectively, and heterodimer (C) **3**. Equipment used: Agilent 1100 series. Solvents: (A) 0.1% TFA in H_2O and (B) 0.1% TFA in AcCN, analytical column: Chromolith RP-18e (4.6 mm × 100 mm; Merck, Darmstadt, Germany), gradient used: (A−B) 0−100% B in 6 min, flow: 4 mL/min

Table S1.
MALDI-MS results for compounds **1´-2´** and **3**.

		**Calc.M**	**$[M+H]^+$**
1´	$C_{39}H_{55}N_9O_{10}$	809.92	810.5
2´	$C_{67}H_{96}N_{16}O_{13}$	1333.60	1334.4
3	$C_{122}H_{177}N_{29}O_{30}$	2529.93	2530.3

Radiolabeling

The heterodimer DOTA-RM2-617 and the two monomers, PSMA-617 and DOTA-RM2, were labeled with the PET diagnostic radiometal ^{68}Ga and the therapeutic ^{177}Lu.

^{68}Ga labeling: Briefly (0.3−1.0 nmol) of each precursor in 0.1 M HEPES buffer, (pH = 7.5, 100 µL), were incubated with a mixture of HEPES buffer (2.1 M, 10 µL) and 40 µL (80−100 MBq) ^{68}Ga (eluted from a ^{68}Ge/^{68}Ga generator based on pyrogallol resin support as [^{68}Ga]GaCl$_4$). The pH of the labeling solution was adjusted to pH = 4.2 using 30% NaOH and the reaction mixture was incubated at 98 °C for 15 min.

^{177}Lu labeling: ^{177}Lu was obtained from PerkinElmer as [^{177}Lu]LuCl$_3$ in 0.05 M HCl. To the [^{177}Lu]LuCl$_3$ (5 µL, 20 MBq) an amount of Na-Ac (115 µL, 400nM, pH: 5.0) was added and of 0.1–1 mM of each substance (~5 nmol) the reaction mixture was left to incubate at 98 °C for 25 min. Labeling efficiency in all cases was determined via analytical RP-HPLC. The results of the Analysis are presented in **Figure S2.**

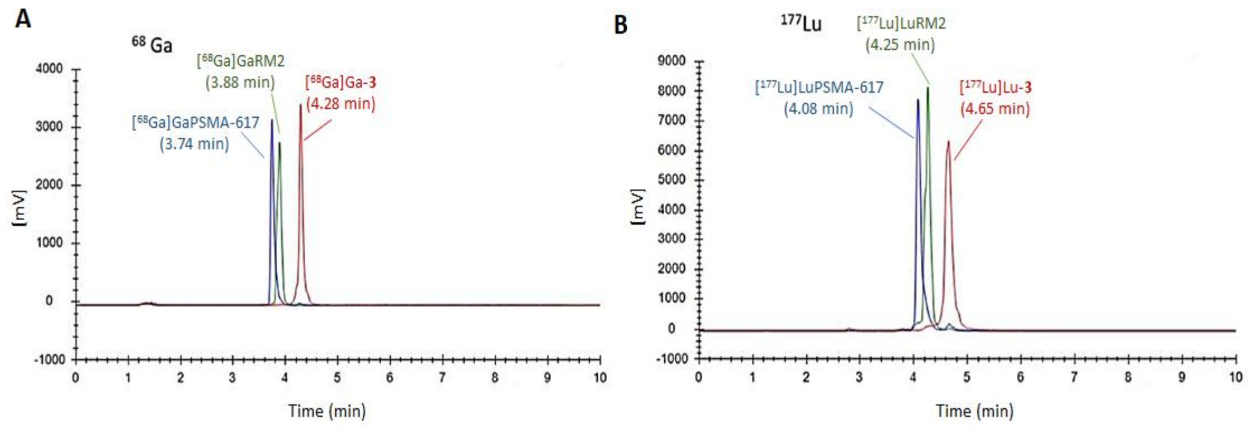

Figure S2.
Comparative radio RP-HPLC of (A) [^{68}Ga]Ga-**3** and reference compounds [^{68}Ga]Ga-PSMA-617, [^{68}Ga]Ga-RM2 and (B) [^{177}Lu]Lu-**3** and [^{177}Lu]Lu-PSMA-617, [^{177}Lu]Lu-RM2. Equipment used: Agilent 1100 series. Solvents: (A) 0.1% TFA in H_2O and (B) 0.1% TFA in AcCN, analytical column: Chromolith RP-18e (4.6 mm × 100 mm; Merck, Darmstadt, Germany), gradient used: (A−B) 0−100% B in 6 min, flow: 4 mL/min

Determination of lipophilicity

An aliquot of each ^{68}Ga-ligand (20 µL) i.e., [^{68}Ga]GaPSMA-617, [^{68}Ga]GaRM2, [^{68}Ga]Ga-**3** and [^{177}Lu]LuPSMA-617, [^{177}Lu]LuRM2, [^{177}Lu]Lu-**3** was added to a mixture of PBS (pH = 7.4, PAN-Biotech, Aidenbach, Germany) /1-octanol (Allchem GmbH Chemikalienvertrieb, Breisach, Germany) (1:1, v/v, 500 µL and 500 µL), rigorously vortexed and centrifuged (2800 rpm/8 min) and the radioactivity of each phase (aliquot of 40 µl) was measured with a gamma-counter Cobra II (PerkinElmer, Waltham, Massachusetts).

The lipophilicities of all compounds were determined by their equilibrium distribution in the two phases according to the distribution coefficient log D:

$$logD_{n-octanol/PBS} = log\frac{[solute]_{octanol}^{ionized} + [solute]_{octanol}^{nonionized}}{[solute]_{PBS}^{ionized} + [solute]_{PBS}^{nonionized}}$$

where:

$[solute]_{octanol}^{ionized}$ is the concentration of ionized compound in the n-octanol phase and $[solute]_{octanol}^{nonionized}$ of the nonionized ligand in n-octanol phase, and $[solute]_{PBS}^{ionized}$ is the concentration of ionized compound in PBS, and $[solute]_{PBS}^{nonionized}$ is the concentration of nonionized ligand in PBS. Each measurement was performed in triplicates and results were expressed as Means ± Standard deviation. A summary of the results is presented in **Table S2**.

Table S2.
Distribution coefficient logD in n-octanol/PBS (pH = 7.4) for [^{177}Lu]Lu-PSMA-617, [^{68}Ga]Ga-RM2, [^{68}Ga]Ga-3 and [^{177}Lu]Lu-PSMA-617, [^{177}Lu]Lu-RM2, [^{177}Lu]Lu-3.

Radionuclide used in the complex	logD [a]		
	PSMA-617 (1)	RM2 (2)	3
[^{68}Ga]Ga	-2.99 ± 0.05	-2.93 ± 0.1	-1.82 ± 0.01
[^{177}Lu]Lu	-3.41 ± 0.50	-3.01 ± 0.08	-2.45 ± 0.01

[a] determined as mean from 3 repeats

Biological testing

Cell Culture & cell assays

In vitro and *in vivo* experiments were performed with the following cell lines: the GRPR positive PC-3 cells (bone metastasis of a grade IV prostatic adenocarcinoma, ATCC CRL- 1435) and the PSMA-positive LNCaP cells (ATCC CRL-1740), which were cultured in DMEM and RPMI 1640 medium, respectively supplemented with 10% fetal calf serum and 2 mM L-glutamine (Invitrogen, Carlsbad, CA, USA). Cells were grown at 37 °C in humidified air with 5% CO_2. Trypsin-ethylenediaminetetraacetic acid (trypsin-EDTA; 0.25% trypsin, 0.02% EDTA, Invitrogen) was used for cell harvesting.

Determination of Binding Affinity in LNCaP and PC-3 cells

The assays were performed according to previously described methods.[3-5] Briefly, LNCaP 10^5 cells per well were incubated with a solution of ^{68}Ga-labeled radioligand [Glu-urea-Lys(Ahx)]$_2$-HBED-CC (0.75 nM, PSMA-10, precursor purchased from ABX, Radeberg, Germany) and an ascending series of 12 different analyte concentrations (0, 0.5, 1, 2.5, 5, 10, 25, 50, 100, 500, 1000 and 5000 nM, 100 µL/well in Optimem,

45 min). This mixture was removed after incubation and the wells were washed on a multiscreen vacuum manifold (Millipore, Billerica, MA) three times with PBS. The following detection of cell-bound radioactivity was measured using a gamma counter (Packard Cobra II, GMI, Minnesota, USA). Data was fitted with a nonlinear regression algorithm (GraphPad Software) to determine the 50% inhibitory concentration (IC_{50}) values.

A similar procedure was followed for PC-3 cells.[4] A population of 10^5 PC-3 cells /well was incubated with ^{125}I-[Tyr^4]-BN ([^{125}I]I-BBN C = 50 pM, 722kBq (19,4μCi), 72,2GBq/μmol, Perkin Elmer) and an ascending series of 8 different analyte (**3**) concentrations (0, 0.025, 0.25, 2.5, 25, 125, 250, 1250, 12500 nM) for 45 min. After incubation the mixture was removed, the wells were washed with PBS and the cell-bound radioactivity was measured using a gamma counter (Hidex AMG - Automated Gamma Counter). Data was fitted with a nonlinear regression algorithm (GraphPad Software) to determine the 50% inhibitory concentration (IC_{50}) values. The results expressed as IC_{50} (nM) values, for the heterodimer **3** and the respective monomers **1** and **2**, (reference compounds) are summarized in **Figure 2** and **Table S3**, along with literature data (expressed as IC_{50} or K_i values in nM). Differences between the literature affinity values regarding the reference compounds are due to the different experimental setups and conditions.

Table S3.
Binding affinities IC_{50} (nM)* or Ki (nM)$^{\$}$ values of heterodimer **3** and controls **1** (PSMA-617 pharmacophore) and **2** (RM2 pharmacophore) determined against PSMA receptor in LNCaP cells (PSMA+, GRPR-) and GRPR in PC-3 cells (PSMA-, GRPR+).

Compound	LNCaP	PC-3	Ref
3	21.41*	43.93*	-
2 (RM2 pharmacophore)	-	45.59*	-
RM2		7.7	[1]
1 (PSMA-617 pharmacophore)	6.41*	-	-
PSMA-617	2.34$^{\$}$	-	[2]

Time Kinetic Cell Binding

Specificity of binding in relation with time was analyzed using a modified protocol[5]. Solutions of the ^{68}Ga-labeled compounds (30 nM, 30 MBq/nmol), were added to 1 × 10^6 cells (LNCaP or PC-3) suspended in 0.1 mL Opti-MEM (Gibco, Auckland, New Zealand) and incubated at 37 °C. Samples were briefly vortexed and a 10 μL aliquot (1-1.4 × 10^5 cells) was taken at predetermined time points: 15, 30, 45, 60, 90 min. The aliquot was then transferred to a 400 μL microcentrifuge tube (Roth, Germany) containing 350 μL of a 75:25 mixture of silicon oil, density 1.05 (Sigma Aldrich, Germany), and mineral oil, density 0.872 (Acros, Nidderau, Germany). Separation of cells from the medium was performed by centrifugation at 12000 rpm for 2 min. After freezing the tubes using liquid nitrogen, the bottom tips containing the cell pellet were cut off. The cell pellets and the supernatants were separately counted in a γ counter. Non-specific binding was

determined by adding a 1000-fold excess of a blocking substance BN or H_2N-PEG_2-4-amino-1-carboxymethylpiperidine-[(R)-Phe^6,Sta^{13},Leu^{14}]-BN(6–14), 100 mM solution in DMSO. Cell binding (cell counts) was determined as the percentage of the total counts added to the cell suspension (counts for tip and top). The results are presented in **Figure S3**.

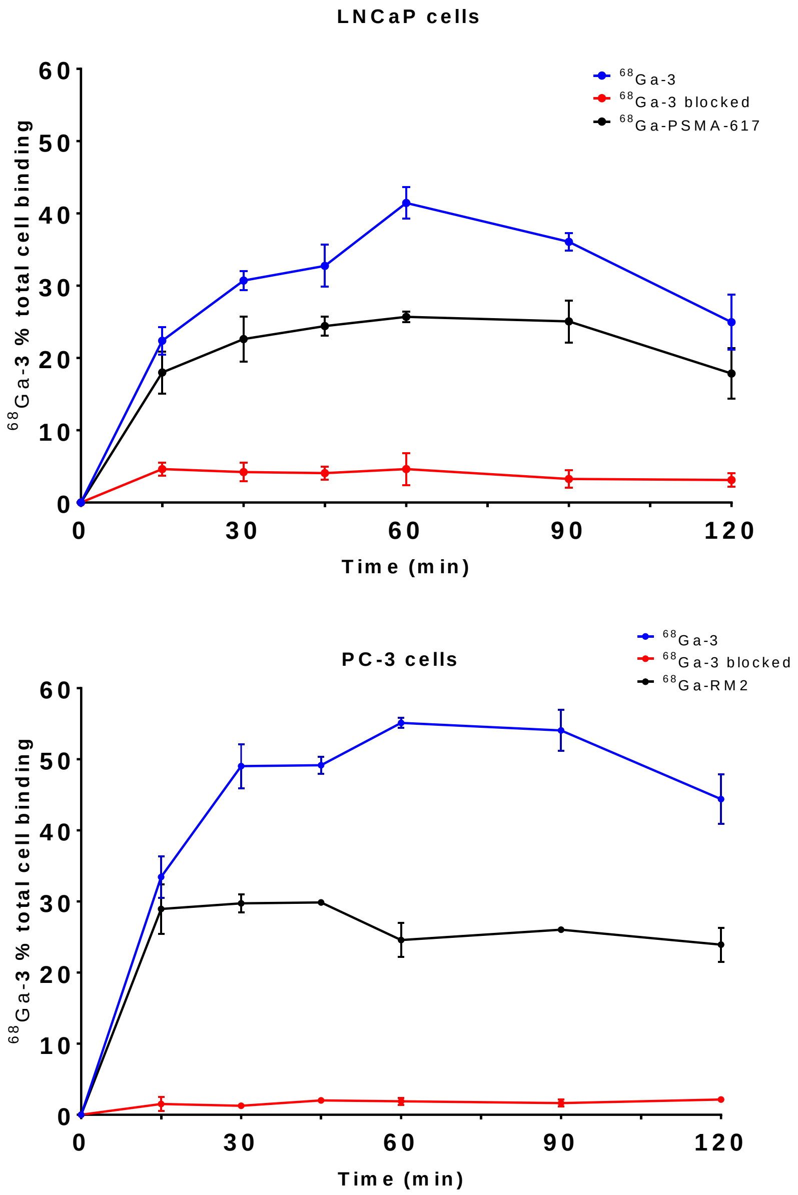

Figure S3

Total cell-bound radioactivity over time, expressed as % of the added (Mean value ± SD, N=3) [^{68}Ga]Ga-**3** (blue) (30 nM, 30 MBq/nmol); blocked [^{68}Ga]Ga-**3** (red) (with 1000-fold excess of PMPA for LNCaP, and BN for PC-3); controls [^{68}Ga]Ga-PSMA-617 for LNCaP cells and [^{68}Ga]Ga-RM2 for PC-3 cells.

Internalization Experiments in PC-3 and LNCaP Cells

Internalization experiments were performed as previously described.[5] Briefly, 10^6 PC-3 or LNCaP cells were seeded in 6-well cell culture plates 24 h before the day of the experiment. The cells were incubated with the radiolabeled compounds (30 nM, in reduced serum, Opti-MEM, Gibco) for 45 min at 37 and 4 °C, respectively. To determine specific cellular uptake, cells were blocked by competition with 1000-fold excess of GRPR block: H_2N-PEG_2-4-amino-1-carboxymethylpiperidine-[D-Phe^6, Sta^{13},Leu^{14}]-BN(6−14) (C = 30 μM/Well) and PSMA block: 2-PMPA (2-(phosphonomethyl)pentane-1,5-dioic acid) (C = 500 μM/well). After incubation the supernatant was removed and the cells were washed with ice-cold PBS. To remove surface-bound radioactivity, cells were incubated twice with 0.5 mL of glycine-HCl in PBS (50 mM, pH 2.8) for 5 min. Then cells were washed with 1.0 mL of ice-cold PBS and lysed using 0.5 mL of 0.3 N NaOH (internalized radioactivity). The surface-bound and the internalized fraction were measured in a γ counter (Packard Cobra II, GMI, Ramsey, MN, USA).

Biodistribution

Tumor xenographs were created by subcutaneously inoculation of 5×10^6 cells of PC-3 or LNCaP (in 50% Matrigel; Becton Dickinson, Heidelberg, Germany) into the right trunk of 7- to 8-week-old SCID mice to obtain the experimental xenograft tumor models. Tumors were allowed to grow to approximately 300 mm^3 in size. Each of the ^{68}Ga-labeled compounds was injected into a tail vein (1−2 MBq; 60 pmol). Syringes were measured before and after administration and appropriate corrections were made for the calculation of the injected dose (ID). The animals were euthanized by isofluorane inhalation at predetermined time points (30 min, 60 min and 90 min) post injection. The dissected major organs along with blood and muscle samples were excised and measured. The radioactivity uptake was measured with a gamma counter and calculated as % ID/g. The % IA in whole blood was estimated assuming a whole blood volume of 6.5% of the total body weight. All animal experiments complied with the current laws of the EU.

Docking calculations

For the wild type truncated PSMA protein simulations, the crystal structure PDB ID 3D7H [(6)] (the truncated hGCPII complexed to the Glu-urea-Lys containing inhibitor DCIBzL) was used as the induced fit docking method for modeling the binding of the PSMA-617 part of **3** (the Glide program was available in Schrödinger 2017-1 platform - Schrödinger Release 2021-1: Glide, Schrödinger, LLC, New York, NY, 2021) [(7)]. A maximum of 20 poses was applied and we chose the highest-scored docking pose (Figure 1).

The cryo-EM structure (PDB ID 7W40)[8] of GRPR in complex with Gαq and the peptide-agonist [D-Phe^6, β-Ala^{11}, Phe^{13}, Nle^{14})Bn(6–14)] was used as template for the docking calculations. All residues of the peptide-agonist ligand [D-Phe^6, β-Ala^{11}, Phe^{13}, Nle^{14})Bn(6–14)] were manually mutated to RM2's amino acid residues and further modifications, e.g., the addition of the linker and the chelator were applied using the 3D-build panel on Maestro software (Schrödinger Release 2020-4: Maestro, Schrödinger, LLC, New York, NY, 2020). The complex was further processed using the Protein Preparation Workflow (Schrödinger Release 2020-4: Maestro, Schrödinger, LLC, New York, NY, 2020). The docking poses of the RM2 part of **3** into the binding area of GRPR were performed using the Monte-Carlo/Low-Mode algorithm as

implemented on MacroModel software (Schrödinger Release 2020-4: MacroModel, Schrödinger, LLC, New York, NY, 2020) previously described also in ref.[5] (Figure 1)

References

(1) Mansi, R.; et al. *Eur. J. Nucl. Med. Mol. Imaging*. 2011, 38 (1), 97–107. (2) Benešová, et al. *J Nucl Med.* 2015, 56 (6), 914–920. (3) Eder, M et al. *Bioconjug. Chem*. 2012, 23 (4), 688–697. (4) Eder, M. et al. *Pharmaceuticals (Basel).* 2014, 7 (7), 779–796. (5) Liolios, C et al. *Mol. Pharm.* 2022. (6) Barinka, C., et al. *J Med Chem.* 2008, *51* (24), 7737–7743, (7) Friesner, R. A.; et al. J Med Chem 2004, 47 (7), 1739–1749. (8) Peng, S.; et al. *Proc. Natl. Acad. Sci*. *U.S.A.* 2023, 120
